# Parametric Rule-Based Intelligent System (PRISM) for Design and Analysis of High-Strength Separable Microneedles

**DOI:** 10.3390/mi16070726

**Published:** 2025-06-21

**Authors:** Sanghwi Ju, Seung-hyun Im, Kyungsun Seo, Junhyeok Lee, Seokjae Kim, Tongil Park, Taeksu Lee, Byungjeon Kang, Jayoung Kim, Ryong Sung, Jong-Oh Park, Doyeon Bang

**Affiliations:** 1 Graduate School of Data Science, Chonnam National University, 77 Yongbong-ro, Buk-gu, Gwangju 61186, Republic of Korea; shju1233@kimiro.re.kr (S.J.); bjkang8204@jnu.ac.kr (B.K.); 2 Robot Research Initiative, Chonnam National University, 77 Yongbong-ro, Buk-gu, Gwangju 61186, Republic of Korea; 3 Korea Institute of Medical Microrobotics, 43–26, Cheomdangwagi-ro 208-beon-gil, Buk-gu, Gwangju 61011, Republic of Korea; tmdguszza@kimiro.re.kr (S.-h.I.); 2jhyeok@kimiro.re.kr (J.L.); outcry9@kimiro.re.kr (S.K.); tongil.park@kimiro.re.kr (T.P.); tslee@kimiro.re.kr (T.L.); jaya@cbnu.ac.kr (J.K.); 4 Fint Korea Inc., 60–7 Nanosandan 5-ro, Nam-myeon, Jangseong-gun 57248, Republic of Korea; nssks3@naver.com; 5 Department of AI Convergence, Chonnam National University, 77 Yongbong-ro, Buk-gu, Gwangju 61186, Republic of Korea; 6 Department of Biosystems Engineering, Chungbuk National University, Cheongju 28644, Republic of Korea

**Keywords:** separable microneedles, parametric design, micro-3D printing, mechanical strength, drug delivery

## Abstract

Transdermal microneedle systems have received great attention due to their minimally invasive way of delivering biomolecules through the skin with reduced pain. However, designing high-strength separable microneedles, which enable easy skin penetration and easy patch detachment, is challenging. Here, we present a Parametric Rule-based Intelligent System (PRISM), which generates the design of and analyzes high-strength separable microneedles. The PRISM platform integrates parametric 3D modeling, geometry-based structural analysis, and high-resolution micro-3D printing for the creation of high-strength separable microneedles. We fabricated prototype microneedle arrays via microscale stereolithographic printing (pµSL) and demonstrated separation of microneedle tips in a skin-mimicking phantom sample. Mechanical testing showed that the suggested design achieved 2.13 ± 0.51 N axial resistance and 73.92 ± 34.77 mN shear fracture force; this surpasses that of conventional designs. Finally, an experiment using a skin-mimicking artificial phantom sample confirmed that only the PRISM-designed separable microneedles could have been inserted and separated at the target depth, whereas conventional designs failed to detach. This approach addresses the development of microneedle systems, which achieve both robust skin phantom penetration and reliable separable delivery, presenting an efficient development tool in transdermal drug delivery technology.

## 1. Introduction

Transdermal microneedles have gained great attention due to their minimally invasive biomolecular delivery in comparison with conventional hypodermic injections. Therefore, biomolecular delivery, such as drugs or vaccines by using microneedles, is advantageous to patients by avoiding pain and bleeding, because microneedles could deliver drugs or vaccines through the skin without reaching nerves or blood vessels [1]. Thus, various types of microneedles have been developed, including solid microneedles, dissolving microneedles, and hollow microneedles. Among these reports, separable microneedles are of particular interest for sustained delivery because the microneedle tips are separated from the substrate and remain embedded as a depot that can release drugs over time after insertion into the skin [2,3]. For example, separable arrowhead microneedles consisting of polymer drug-loaded tips on metal shafts were developed [2]. After insertion, the sharp polymer tips are detached from the substrate and embedded in the skin. Also, there is another example, where needle tips made of polyvinyl alcohol (PVA) were mounted on the substrate. After insertion, a reaction generated gas bubbles, which rapidly separated the tips within ~90 s [3]. Stimuli-responsive separating microneedle designs have also been reported, such as microneedles, which separate upon exposure to near-infrared light or heat [4].

Despite these examples exhibiting capabilities of the application of microneedles, engineering a separable microneedle which is both mechanically robust and reliably detachable remains a key challenge [5,6]. The microneedles need to have endurance against high axial force to penetrate the skin without breaking prematurely [7]. Also, at the same time, the tip can be easily separated from the substrate by applying shear force to ensure microneedle tips are embedded after the substrate is removed from the skin [8,9,10]. Therefore, achieving an optimal balance between high axial strength and low shear fracture force is crucial for the development of robust high-strength separable microneedles [11]. Previous separable microneedle solutions are generally based on multi-step fabrication with various components, which results in complicated manufacturing processes, as well as limited design. Thus, there is a need for a systematic design approach to optimize microneedle geometry for both penetration strength and microneedle tip separation, which is based on a single structural material and an automated precise 3D design and fabrication process.

**Figure 1:**
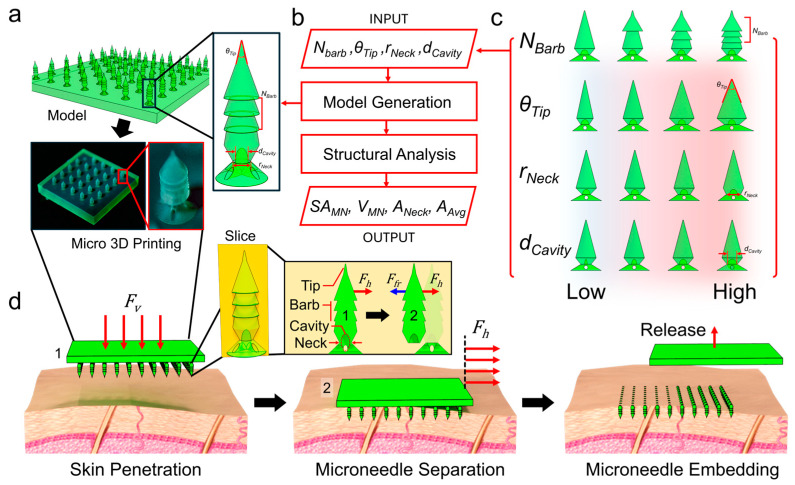
Concept of Parametric Rule-based Intelligent System (PRISM) for design and analysis of high-strength separable microneedles. (**a**) PRISM generates a 3D model of high-strength separable microneedle arrays with respect to input parameters. Then, a high-strength separable microneedle array is fabricated by using micro-3D printing. The inset (top right) describes parameters for a microneedle design. (**b**) A simplified schematic flowchart of the PRISM workflow. Users input parameters (*N_Barb_*, *θ_Tip_*, *r_Neck_*, *d_Cavity_*) and the PRISM platform automatically generates the 3D microneedle model and performs structural analysis based on the 3D model (*SA_MN_*, *V_MN_*, *A_Neck_*, *A_Avg_*). These output parameters are related to drug release speed, drug loading capacity, and mechanical strength for penetration and separation. (**c**) A schematic illustration of the 3D model structure according to the change in each input parameter. (**d**) A schematic illustration of the application of high-strength separable microneedles. At first, the microneedle array is pressed on the skin using a vertical force *F_v_* (red arrow) to insert the microneedles into the skin phantom. Then, a horizontal force *F_h_* (red arrow) is applied by sliding the sample. This results in the microneedles fracturing at their necks. After separation, the sample is removed, leaving the microneedle tips embedded in the skin phantom.

Here, we address these challenges by developing a Parametric Rule-based Intelligent System (PRISM) for the design and analysis of high-strength separable microneedles (Figure 1). The PRISM platform allows researchers to rapidly generate 3D microneedle models with tunable geometric features and automatically evaluate their structural characteristics based on their 3D microneedle model. Input parameters (features) include the number of barbs on the needle (*N_Barb_*), the tip angle (*θ_Tip_*), the neck diameter ratio (*r_Neck_*), and the size of an internal cavity at the neck (*d_Cavity_*). By adjusting these features, PRISM computes output metrics (labels) such as the needle’s total surface area, which is related to drug release flux, volume, which is related to drug loading capacity, average cross-sectional area, which is related to axial hardness for penetration, and cross-sectional area at the neck, which is related to easiness for microneedle tip separation. We hypothesized that our parametric approach could result in design improvement, which improves the ratio of axial strength to fracture force, which means that the needles penetrate effectively yet separate easily.

To validate the performance of the microneedles generated by PRISM, we fabricated prototype microneedle arrays using high-resolution microstereolithography (pµSL). This technique has the capability to produce complicated 3D microscale structures with feature sizes of micrometers and therefore can enable the realization of PRISM-generated microscale 3D designs, such as barbed tips and internal cavities. Thereafter, we validated the mechanical characterization on the 3D-printed microneedles to measure their vertical (axial) compression strength and horizontal shear fracture force, simulating the forces experienced during insertion and separation. Finally, we conducted insertion experiments using skin-mimicking phantom materials to demonstrate that the PRISM-generated separable microneedles can successfully embed microneedle tips in the sample. The results support that our PRISM-generated microneedles achieve a unique combination of robustness and separability, outperforming conventional microneedle geometries in tip detachment efficacy.

## 2. Materials and Methods

### 2.1. Materials

Microneedles were fabricated using a biocompatible light-sensitive resin (BMF MED; BMF Precision Tech Inc., Shenzhen, China) optimized for high-resolution digital light processing (DLP) 3D printing. The resin was selected for its biocompatibility and suitability for skin-interfacing biomedical applications. Parafilm M (Bemis, Neenah, WI, USA) was used to fabricate artificial skin phantoms. Isopropyl alcohol (IPA, Duksan General Science, Seoul, Republic of Korea) was used for post-curing and cleaning. All materials were used without further purification.

### 2.2. Methods

#### 2.2.1. Parametric Design and Modeling

**Figure 2:**
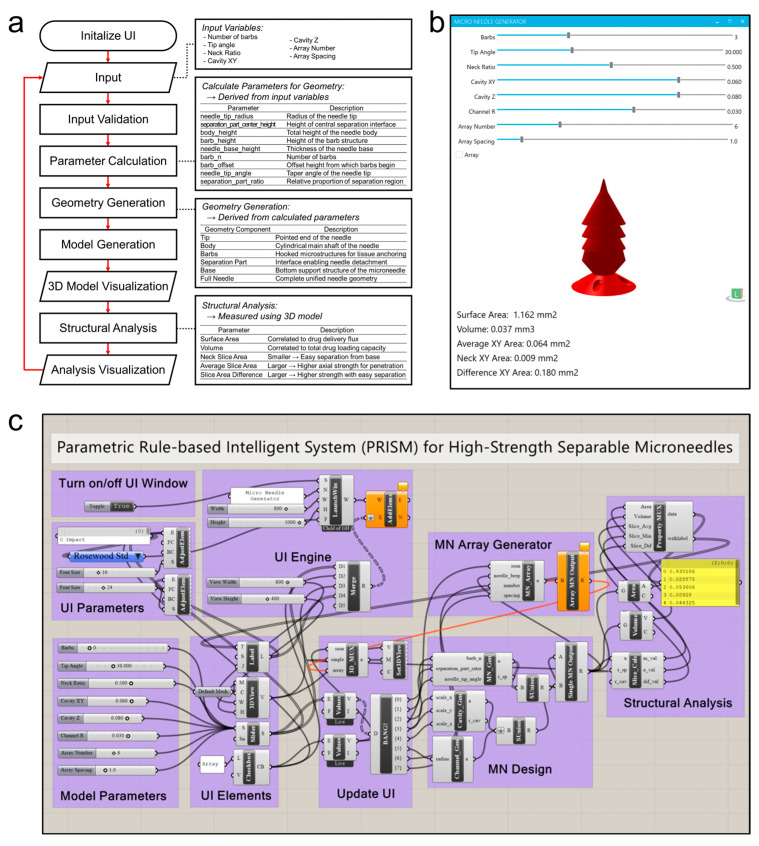
PRISM software (Rhinoceros 3D version 8 SR20; Washington, Seattle, USA), (Grasshopper version 1.0.0008; Washington, Seattle, USA) workflow and implementation. (**a**) A flowchart of the PRISM algorithm. After initializing the graphical user interface (GUI), the user inputs the microneedle design parameters. Then, the PRISM algorithm validates inputs, calculates geometric parameters (e.g., actual lengths for tip, barb, or base), and the 3D microneedle model is generated. Then, the structural analysis module calculates output parameters, such as surface area, volume, cross-sectional area at neck, and average cross-sectional area. Finally, the visualization step provides these outputs (3D model and structure analysis parameters) to the user by using GUI. (**b**) A screenshot of the PRISM software GUI. The interface features input for each microneedle generation parameter, allowing the user to set values of the parameters and receive results in real time. (**c**) A schematic illustration of the parametric design logic (Grasshopper for Rhinoceros 3D).

PRISM (Parametric Rule-based Intelligent System) is a computational system that is designed to automate the production of structurally enhanced separable microneedles (Figure 2). PRISM was developed with Rhinoceros 3D (version 8 SR20; Seattle, WA, USA) and its visual programming interface, Grasshopper (version 1.0.0008; Seattle, WA, USA), which comprises modular components for parameter input, geometry construction, structural analysis, and real-time visualization. By using the user interface (GUI), PRISM allows users to dynamically change important microneedle design features, including tip angle, barb count, narrowed neck ratio, and cavity dimensions. Once data have been entered, PRISM computes secondary geometric features, including neck diameter, shaft height, barb height, and barb spacing, by automatically verifying the input. These details then enable an all-encompassing 3D microneedle model with structural features including the internal cavity, tapered shaft, narrower neck, and barbed zone. The main measurements related to shape that PRISM’s structural analysis module provides include volume, surface area, average cross-sectional area, and the smallest cross-sectional area at the neck. PRISM’s integrated structural analysis module allows users to predict the expected performance before actually fabricating the structure. Volume, surface area, average cross-sectional area, and minimum cross-sectional area at the neck are computed, and the mechanical performance of the microneedles is projected using these data, including axial resistance and shear separability. The produced 3D model and output values shown in the GUI together provide real-time feedback and iterative design development (Figure 2b).

This tool enables users to graphically evaluate geometric outputs, interactively modify design parameters, and maximize design performance before committing to manufacture. Grasshopper’s modularization of the entire PRISM process allows for the scalable and reusable deployment of future microneedle versions (Figure 2c). The 3d designs created by Rhinoceros 3D-grasshopper are then exported as STL files and fabricated by using projection microstereolithography (pµSL) with a slicing resolution of 5 μm with exceptional geometric fidelity.

In this study, after setting the upper diameter of the upper body of the microneedle and the barb structure to 10 µm, Equation (S1) was used to calculate the bottom radius of the microneedle body based on the tip angle to create a tapered structure that becomes the upper body of the microneedle. Equation (S2) was used to determine the diameter of the narrated neck at the bottom radius of the structure to create a candle shape.

Supplementary Note S1 provides a detailed description of the parametric design logic. Each equation describes the geometric relations employed by PRISM to calculate the microneedle upper body’s tip angle, narrow neck radius, and barb designs. These equations enable high fidelity structural specification for microneedle fabrication.

In this study, we constructed four sets of microneedle samples to evaluate various structural aspects of each microneedle group according to their mechanical properties. As Table 1 shows, in all experiments, Group B with a narrow neck of 50% diameter was used as a control group, and Group A was tested with a larger neck (62.5% of the microneedle upper body’s bottom diameter) to investigate how the neck thickness affected the structural strength. Group C integrated the internal cavity into the control configuration (Group B) to see how they affected the compression and shear performance. Finally, Group D integrated three barb structures into the design of Group C to evaluate the effect of external barb structures on mechanical resistance. For the same experimental conditions, the upper body tip angle of the microneedle was fixed at 30 degrees.

#### 2.2.2. Microneedle Fabrication

Microneedles were manufactured using a commercial DLP 3D printer (microArch S230; BMF Precision Tech Inc., Shenzhen, China) with a 5 μm slicing resolution, matching the thickness of each printed layer. The slicing procedure was carried out using VoxelDance Additive software (version 4.1.12.48; Shanghai, China), which is compatible with the BMF printing system. Because of the high viscosity of BMF MED resin at room temperature, it can limit smooth resin flow and homogeneous coating of each 5 μm layer throughout the printing process. To overcome this constraint and achieve consistent layer production, the resin bath was heated to 45 °C.

Samples were printed immediately on the BMF s230’s printing platform. After printing, the entire platform was immersed in isopropyl alcohol to allow the first wash to be performed for 15 min, then the platform was removed from the isopropyl solution and placed on a hot plate to heat the platform. The platform was heated to 60 °C for 6 min to soften the interface between printed samples and the platform surface. After heating the platform for 6 min, the samples were gently detached from the platform using a plastic razor blade, minimizing structural damage to delicate features such as the tip or barbs. The detached microneedle array samples were put in a fresh IPA solution and gently mixed. To guarantee complete polymerization, the final curing process was carried out in a uv oven (Form Cure 2nd Generation; Formlabs Inc., Somerville, MA, USA) at 60 °C for 60 min.

#### 2.2.3. Dimensional Characterization

Cross-sectional images of 1 × 4 linear microneedle arrays (*n* = 4 per group) were taken by using an upright microscope (Axio Imager.A2; Zeiss, Ltd., Oberkochen, Germany). The samples were compared to their corresponding CAD models to evaluate the geometric fidelity of the generated microneedles. Side-profile photos were used to measure key structural features such as barb diameters, separation zone height (narrowed neck), distance from needle tip to base of first barb, and overall microneedle height. All dimensional measurements for the samples were performed with ImageJ (version 1.54g; Bethesda, MD, USA). We evaluated the dimensional accuracy of all parameters by calculating the group-wise mean and 99% confidence interval (±). To find fabrication accuracy, we calculated the relative error using the following equation:

(1) $$\mathrm{Relative}\;\mathrm{Error}\;(\%)=\left(\frac{\left|\mathrm{Measured}\;\mathrm{Value}-\mathrm{CAD}\;\mathrm{Value}\right|}{\mathrm{CAD}\;\mathrm{Value}}\right)\times 100$$

This approach allowed us to systematically evaluate the geometric deviation between the designed structure and the fabricated structure. When the relative error between the CAD design and the actual output sample was less than 5%, the standard was set to be good for implementation, and the observation results were listed in Table 2.

#### 2.2.4. Mechanical Test

Two kinds of mechanical properties were evaluated for structural anisotropy. First, we applied a force in an orthogonal direction to the microneedle to measure the breakage point to the axial compression, and we performed shear peeling experiments to measure the force to separate the microneedle. Axial compression tests were carried out using a universal tensile tester (AGS-X; Shimadzu, Kyoto, Japan) with a 500-N load cell. For all test samples, a 2 × 2 microneedle array spaced 1.5 mm apart was used, and the microneedle array was compressed vertically at a constant rate until the total displacement reached 400 μm over 3 s. The separation force is defined as the maximum force measured before a structural defect occurs. Each group was tested with five replicates (*n* = 5).

Shear detachment tests were operated using the microtester (G2; CellScale, Ontario, Waterloo, Canada) to apply shear force. Test samples were prepared as 1 × 2 microneedle arrays with 1.5 mm spacing and mounted onto a custom-designed cartridge holder to ensure consistent alignment and secure fixation. Lateral shear displacement was applied until the point of detachment at a constant rate over 15 s to operate a 2 mm displacement for each microneedle’s detachment. The peak shear force, defined as the maximum force immediately prior to detachment, was recorded for each sample. Each group was tested with five replicates (*n* = 5).

Different specifications of microneedle array samples were used according to the requirements of each test. For axial compression tests, a preliminary test with a single microneedle caused lateral slip during load, which made the force measurement unstable. In addition, a 2 × 2 array with 1 mm spacing caused mechanical interference between adjacent microneedles during compression, resulting in structural collisions. To handle these issues, a 2 × 2 array with 1.5 mm spacing was adopted to ensure mechanical stability and accurate measurement under vertical loads.

For shear compression tests, the microtester (G2; CellScale, Waterloo, ON, Canada) was used. It was applicable for high-resolution measurements of single microneedle structures. Samples were prepared in a 1 × 2 arrangement with 1.5 mm to enable two independent shear fracture measurements per sample while maintaining alignment and fixation to fit the measurement protocol of the device.

#### 2.2.5. Skin Phantom-Based Penetration–Separation Test

To examine the effects of barb structures on insertion performance and shear-directional separability, penetration–separation tests were performed on Group B and Group D using artificial skin phantoms composed of eight stacked layers of Parafilm. This multilayer configuration simulates the mechanical resistance of human skin and allows for a quantitative assessment of microneedle behavior during both insertion and detachment. Skin phantom experiments were performed using samples fabricated with microneedle arrays at 500 μm intervals to mimic the specifications used in a previous study [12]. Each array was vertically inserted into the skin phantom using a universal tensile tester (AGS-X; Shimadzu, Japan). The axial compression was constantly applied at a rate of traveling 2 mm over 15 s and stopped immediately upon reaching the target force of 32 N, which was selected based on the prior literature demonstrating that this magnitude ensures consistent microneedle insertion [13]. Subsequently, a shear-directional displacement of 5 mm was applied at the same speed to simulate detachment. Each sample was tested in five replicates (*n* = 5) to ensure reproducibility. After testing, the skin phantom layers were carefully separated and examined under an upright microscope (Axio Imager.A2; Zeiss, Ltd., Oberkochen, Germany). Puncture marks were classified as holes (clean perforations), tears (elongated ruptures), or dents (indentations without full penetration). Only holes and tears were considered successful insertions. By counting the number of penetration layers for each sample and the number of microneedles remaining in the skin phantom, the effect of cavity and barb structure integration on insertion depth and separation performance was compared and analyzed.

#### 2.2.6. Statistical Analysis

Statistical analyses were performed using Python (SciPy v1.10; statsmodels v0.14). The Shapiro–Wilk test was used to assess data normality. For multi-group comparisons, one-way ANOVA followed by Tukey’s HSD post hoc test was applied. For pairwise comparisons, Welch’s *t*-test and Cohen’s d were used. A significance threshold of *p* < 0.01 was adopted. All graphs were generated using Matplotlib (v3.7), including bar plots with 99% confidence intervals and annotated significance markers.

## 3. Results and Discussion

### 3.1. Structural Analysis of PRISM-Generated Microneedles

In this study, microneedle array samples were fabricated with commercial resin. However, the final goal of this design generation platform is the fabrication of dissolvable microneedles, so we assumed that the proposed design uses directly dissolvable drug delivery materials; on this occasion, we thought that this design platform could be used to roughly predict drug loading performance.

**Figure 3:**
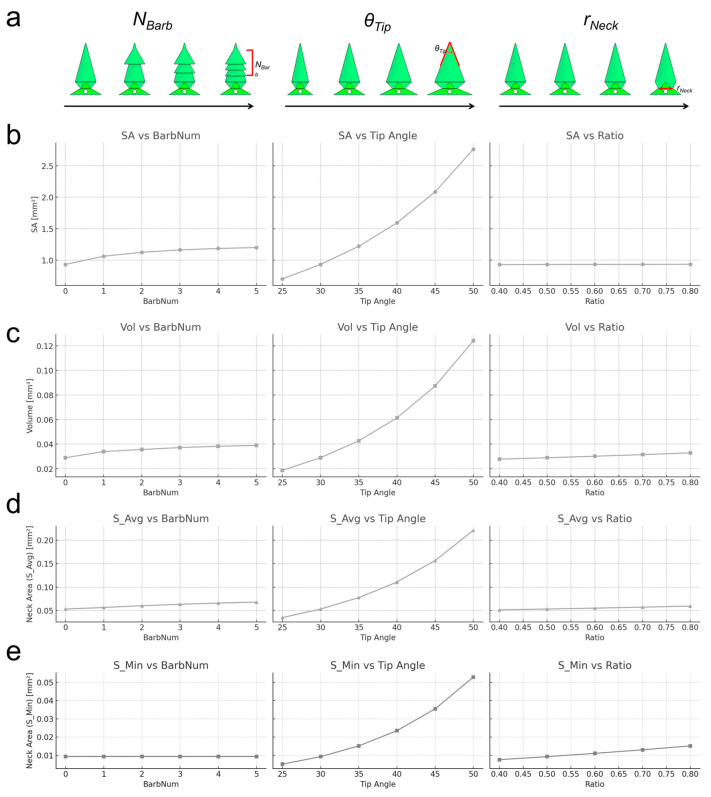
The effect of the input parameters on 3D microneedle structure analysis. (**a**) Schematic illustrations of a 3D microneedle structure depending on the input parameters: number of barbs (*N_Barb_,*), tip angle (*θ_Tip_*), and neck ratio (*r_Neck_*). These parameters correspond to (**b**–**e**). (**b**) The dependency of the surface area (SA), which is correlated to drug delivery flux, is plotted with respect to the change in each input parameter. (**c**) The dependency of the volume (Vol), which is correlated to total drug loading capacity, is plotted with respect to the change in each input parameter. (**d**) The dependency of the average cross-sectional area (S_Avg), in which larger value means higher axial strength for penetration, is plotted with respect to the change in each input parameter. (**e**) The dependency of the neck cross-sectional area (S_Min), in which larger value means higher strength with easy separation, is plotted with respect to the change in each input parameter.

Using the PRISM platform, we quantified how each design parameter influences the microneedles’ structural metrics and performance (Figure 3). We defined four parameters, which are related to the performance of the high-strength separable microneedles with respect to the 3D design of microneedles: surface area (SA), which is correlated to drug delivery flux (Figure 3b), volume (Vol), which is correlated to total drug loading capacity (Figure 3c), average cross-sectional area (S_Avg), in which a larger value means higher axial strength for penetration (Figure 3d), and the neck cross-sectional area (S_Min), in which a lower value means lower strength for separation (Figure 3e).

In general, increasing the number of barbs (*N_Barb_*) on the needle substantially increases the surface area and volume of the needle (since barbs add extra material and surface), which is advantageous for drug delivery capacity. A higher *N_Barb_* also increases *S_Avg* (more structural support along the shaft) but does not change *S_Min* (the neck area) since the neck was defined by *r_Neck_*. The tip angle (*θ_Tip_*) exhibited an inverse relationship with surface area and volume, which means that a smaller tip angle generates a longer needle with more surface area and volume, whereas a larger angle shortens the needle and reduces surface/volume. A smaller tip angle also means a finer point, which can improve penetration ability. The neck diameter ratio (*r_Neck_*) had a strong effect on the cross-sectional areas as lower *r_Neck_* reduces S_Min and reduces S_Avg.

**Figure 4:**
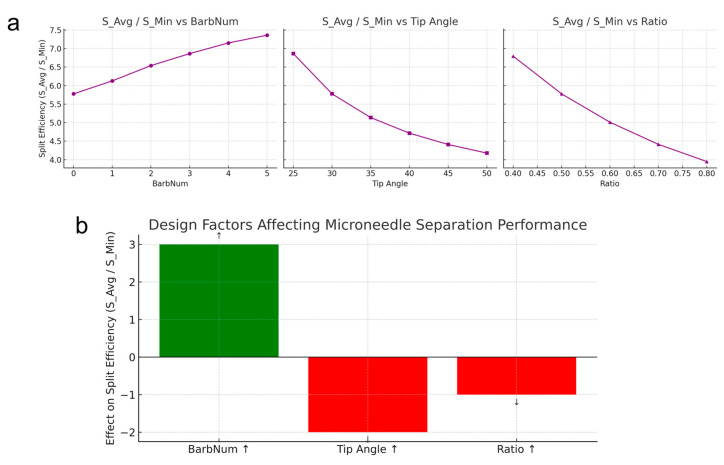
The relationship between the input parameters with microneedle penetration and separation performance. (**a**) Dependencies of the split efficiency, which is defined as S_Avg/S_Min (i.e., average cross-sectional area divided by minimum cross-sectional area at the neck) with respect to the input parameters (BarbNum, Tip Angle, Ratio). This value is high if the microneedle has high strength with high separability. (**b**) A summary of the design factors, which affect microneedle strength and separation performance. The green bars (BarbNum) indicate that increasing these input parameters increases the penetration with split efficiency, while the red bars (Tip Angle and Ratio) indicate that decreasing these input parameters increases the penetration with split efficiency.

Also, we further introduced a composite performance metric “split efficiency”, which is defined as the ratio *S_Avg*/*S_Min* (Figure 4). A high split efficiency indicates a structure which has a large average cross-section relative to a small neck cross-section. Figure 4a depicts the relationship between input parameters with split efficiency. Increasing *N_Barb_* increased the split efficiency (green bar in Figure 4b), whereas decreasing *θ_Tip_* or *r_Neck_* decreased the split efficiency (red bars in Figure 4b). These trends align with intuition and were confirmed by the parametric analyses as more barbs give additional axial support without affecting the neck area, and a thinner neck directly lowers the fracture force.

Additionally, d_cavity is a design feature referring to the bubble structure of previous studies, and structural anisotropy is used to preserve the resistance to vertically applied pressure and to be easily separated even with low shear pressure.

d_cavity insertion will increase the separation performance because the d_cavity inserted structure has a relatively larger proportion of the cross-sectional reduction in the neck structure than the total cross-sectional area reduced due to the d_cavity [14].

### 3.2. PRISM-Generated Microneedle Fabrication and Characterization

**Figure 5:**
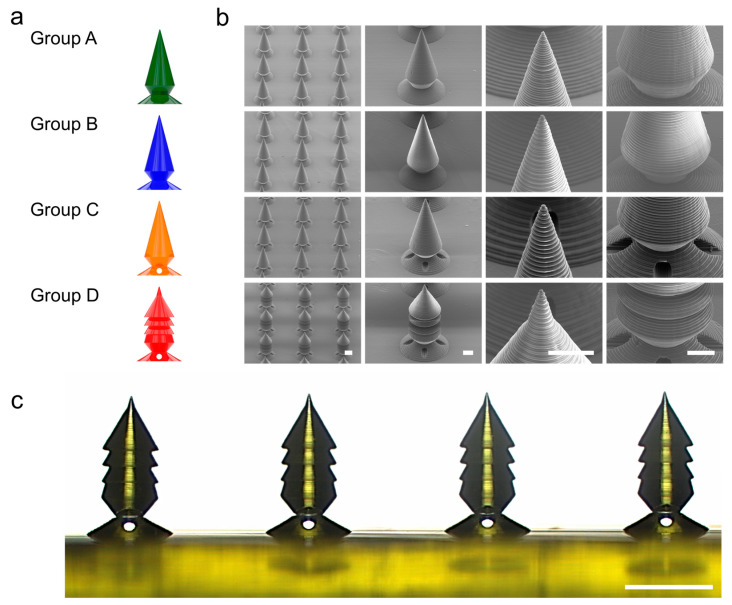
Microscale 3D printing and structural analysis of the PRISM-generated microneedle samples. (**a**) A schematic illustration of the four different microneedle groups (A–D), which was generated by PRISM. Group A (green) is a baseline candlelight design with no barbs and a relatively thick neck (*N_Barb_* = 0, *θ_Tip_* = 30, *r_Neck_* = 62.5, *d_Cavity_* = 0 (No cavity)). Group B (blue) is also a baseline candlelight design with no barbs with a narrower neck (*N_Barb_* = 0, *θ_Tip_* = 30, *r_Neck_* = 50, *d_Cavity_* = 0 (No cavity)). Group C is a separable design with a cavity inside of the candlelight design (*N_Barb_* = 0, *θ_Tip_* = 30, *r_Neck_* = 50, *d_Cavity_* = 0.6). Group D (red) is an optimized design with a multi-barb design with a cavity (*N_Barb_* = 3, *θ_Tip_* = 30, *r_Neck_* = 50, *d_Cavity_* = 0.6). (**b**) Scanning electron microscopy (SEM) images of PRISM-generated microneedle arrays with various magnifications (scale bars are 200 µm in the array images and 100 µm in the single-needle close-ups). (**c**) A side-view optical microscope image of a row of printed microneedles (Group D samples and scale bar is 500 µm).

Figure 5a depicts schematic drawings of the four PRISM-designed microneedle groups (A–D), showing differences in neck thickness, cavity presence, and barb integration. Figure 5b depicts sample SEM (SU8010; HITACHI, Tokyo, Japan) images of printed microneedles, displaying remarkable manufacturing quality at both the array and individual needle levels. Figure 5c shows a side-view optical microscope image of a printed Group D sample, which confirms the geometric clarity of essential features such as the tapering body, narrower neck, and barbed structures.

To assess the geometric fidelity of the final microneedle design (Group D), longitudinal optical microscope photographs of 1 × 4 linear microneedle arrays (*n* = 4 per group) were compared to their matching CAD models. An upright microscope (Axio Imager.A2; Zeiss, Ltd., Oberkochen, Germany) was used to collect side-view images, and ImageJ (version 1.54g; Bethesda, MD, USA) software was used to measure dimensions. Nine structural parameters were examined, including the overall microneedle height, separation zone (narrowed neck) height, distance from the needle tip to the first barb, diameters of the three barbs, maximum diameter before the neck region, narrowed neck diameter, and cavity diameter in the XY plane. Table 2 shows that the measured dimensions are very consistent with the prescribed design values. The microneedle height was 700.07 ± 4.54 µm, with a relative error of 0.01%, consistent with the CAD target of 700 µm. The separation zone height (narrowed neck) was 102.30 ± 2.69 µm, with a 2.30% error, and the distance to the first barb was 325.62 ± 2.57 µm (0.19% error). The barb diameter ranges from 314.03 to 319.08 μm, with accuracy of approximately 96 to 97% compared to the design value of 328 μm. The upper body base diameter showed an accuracy with errors of 1.92% and the narrowed neck diameters showed accuracy with errors of 1.43%, respectively. The cavity diameter in the XY plane was measured to be 119.68 ± 1.35 μm, almost matching the expected value of 120 μm (0.27% error), indicating that the manufacturing of this internal feature was satisfactory. The results show that both external and internal microneedle features are excellent in geometric accuracy and fabrication reproducibility, indicating that structurally complex separable microneedles can be constructed with high fidelity using parametric modeling and DLP 3D printing.

### 3.3. Microneedle Mechanical Test of PRISM-Generated Microneedles

**Figure 6:**
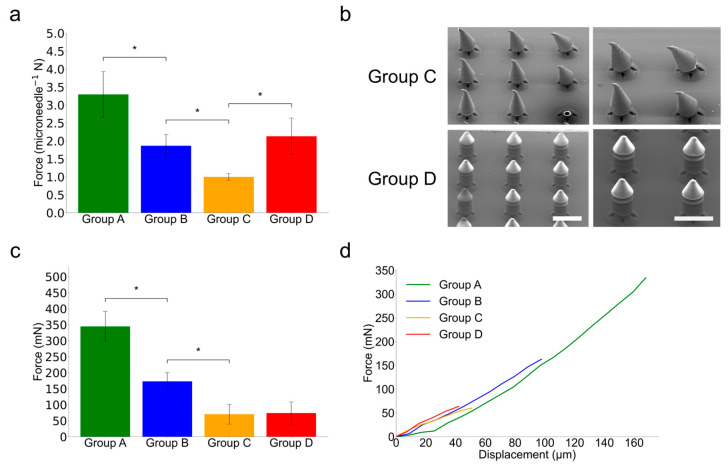
Mechanical testing of PRISM-generated microneedle samples. (**a**) A comparison of peak axial compression resistance for each PRISM-generated microneedle sample groups. The error bars denote standard deviation over five different samples (*n* = 5) and asterisks (*) denote statistically significant differences (*p* < 0.05). (**b**) Scanning electron microscopy (SEM) images of Group C and Group D microneedles after axial compression by 0.1 mm (Scale bar is 500 µm). In Group C, after axial compression by 0.1 mm, the tips of the microneedles are bent due to relatively lower axial structural hardness. However, in Group D, the structures of the microneedles are not changed due to the enhanced structural hardness. (**c**) A comparison of peak shear detachment forces for each PRISM-generated microneedle sample group. The error bars denote standard deviation over five different samples (*n* = 5) and asterisks (*) denote statistically significant differences (*p* < 0.05). The samples are pushed down against a hard surface until they fracture. Group D exhibits comparable peak shear detachment force with Group C and this result suggests that Group D exhibits better skin penetration with similar separability. (**d**) Shear detachment force–displacement curves of each PRISM-generated microneedle sample group.

The mechanical behavior of microneedles was investigated to determine how the external barb structures, internal cavities, and narrowed necks impact the axial compression and shear detachment (Figure 6). Group B served as the control, whereas Group D was the final suggested design (cavity + three barbs). Group A (with a thicker neck, 62.5%) had the highest mean peak axial force (3.29 ± 0.63 N), indicating that neck thickness is important for compressive strength. Group B, which had a smaller neck diameter (50%), showed weaker performance (1.87 ± 0.32 N). Group C showed greater degradation (1.00 ± 0.09 N), indicating the negative impact of the internal cavity on structural integrity. Group D’s use of barbed structures in the cavity-containing design resulted in partial recovery of axial strength (2.13 ± 0.51 N), demonstrating the effectiveness of barbs as mechanical reinforcement. Additionally, the short-stroke compression tests (0.1 mm stroke at 0.133 mm/s) were performed on Groups C and D. Group C samples regularly broke or slanted under stress, whereas Group D samples preserved their shape and only showed localized tip deformation. Through this, we observed that the addition of barb structures with a uniform volume affects the improvement of axial pressure durability.

In the shear compression test in Figure 6c, Group A showed the largest detachment force (344.44 ± 47.17 mN), indicating that the thick neck greatly strengthens shear pressure durability. Group B showed a value of (172.90 ± 27.14 mN), indicating that reducing the diameter of the narrow neck was significant in increasing the shear pressure separation efficiency. Furthermore, Group C with the cavity inserted showed less than half (70.23 ± 31.03 mN) of Group B, and in this regard, we observed that the strategy of introducing a cavity in the narrow neck helps the shear pressure separation strategy. Group D with the barb structure showed improved axial pressure durability in the axial pressure test; however, it shows a similar value (73.92 ± 34.77 mN) to that of Group C (70.23 ± 31.03 mN) in the shear compression test. Force–displacement curves also revealed similar fracture behaviors between Groups C and D, indicating that repeatedly generated barbs in the axial direction do not interfere with shear separation. Our proposal shows that geometric anisotropy can be enhanced through parameter-based design structure adjustment. This feature separation increases design flexibility, enabling axial durability while maintaining lateral separation performance.

### 3.4. Penetration–Separation Test Using Skin-Mimicking Phantom

**Figure 7:**
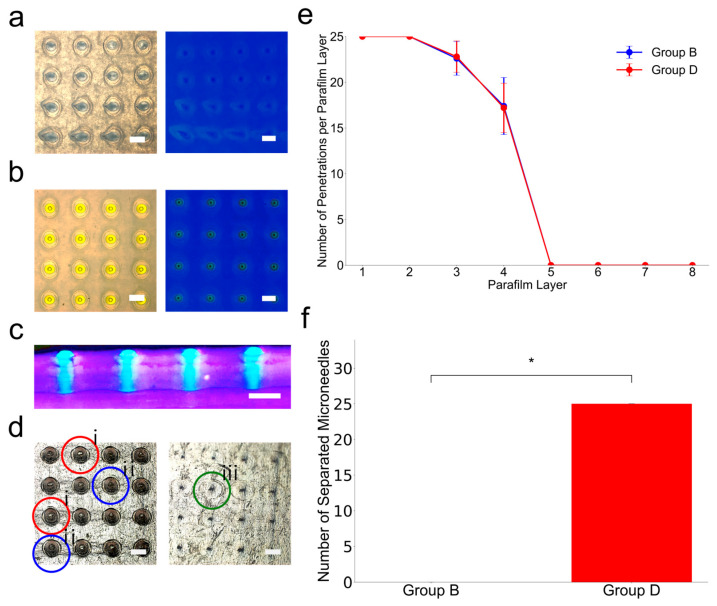
Microneedle insertion and separation tests using skin-mimicking phantom. (**a**) Top-down view of a skin phantom after attempting to insert a microneedle (Group B). In this case, holes are created after microneedle insertion; however, the yellow-colored microneedle is not observed because the microneedles are filed to separation. (**b**) Top-down view of a skin phantom after attempting to insert a microneedle (Group D). In this case, holes and yellow-colored microneedles are observed because the microneedles are separated after applying horizontal force. (**c**) Side cross-sectional view of the skin-mimicking phantom (under UV light) after insertion of the microneedles (Group D). The tips are clearly visible embedded in the skin-mimicking phantom sample, confirming that they have separated from the sample and remain lodged at the sample (Scale bar is 500 µm). (**d**) Bright-field microscopy images of Layer 1 and Layer 6 of the eight layers of parafilm microneedles that were pushed through showing holes, tears, and dents. i. indicates hole, ii. indicates rupture, and iii. indicates dent. (**e**) The number of successful penetrations per parafilm layer for Group B and Group D microneedles (*n* = 5 per group). (**f**) The number of separated microneedles retained within the parafilm skin phantom for Group B and Group D (*n* = 5) and asterisks (*) denote the statistically significant differences (*p* < 0.05).

Penetration–separation experiments were performed on skin phantoms to examine the insertion performance and shear-directional separability of microneedle designs in Groups B and D (Figure 7). Five samples per group (*n* = 5) were evaluated, and the number of penetrated skin phantom layers was counted for each of the eight stacked layers to assess the effects of cavity and barb integration. Figure 7 shows that both groups successfully penetrated the first and second layers. However, the number of penetrations gradually decreased as the layers deepened. In Layer 3, Group B had 22.60 ± 1.84 insertions, while Group D had 22.80 ± 1.72. In Layer 4, insertions dropped further (Group B: 17.40 ± 3.12; Group D: 17.20 ± 2.68), with no microneedles from any group passing beyond the fourth layer. These results demonstrate that the presence of barbs and internal cavities in Group D had no negative effect on the consistency of insertion depth under axial stress.

However, when the shear force was applied after the insertion, a distinct difference was observed in the shear direction separation. The Group B microneedle was not separated from the array, and none of the microneedles remained in the skin phantom. In the process of applying shear force, we observed that the entire microneedle patch escaped from the phantom surface, and the narrowed neck alone had limitations in implementing the separation function. We took a supporting video, video S3, that demonstrated this. Group D showed constant and complete separation in all trials in contrast to Group B. When we performed the same experiment for Group D, for all trials, the Group D microneedle easily separated from the base and remained implanted in the skin phantom. These comparisons highlight that the strategy of introducing the internal cavity structure in the narrowed neck is advantageous for the actual shear separation. The insertion performance was the same throughout the group, but only Group D showed consistent shear-induced separation without damaging the penetration performance.

## 4. Conclusions

In this study, we presented PRISM (Parametric Rule-based Intelligent System) as a novel design and analysis platform for high-strength separable microneedles. By parametrically generating 3D microneedle structures and analyzing their structural characteristics, PRISM enabled the automated generation of a 3D microneedle structure, which has strong axial strength for skin penetration as well as separability. The PRISM-improved design, which has multiple barbs, a narrowed neck, and an internal cavity, was fabricated by using microscale 3D printing. Then, the fabricated PRISM-generated microneedle samples were evaluated for their performance by using a series of mechanical tests and then insertion and separation tests by using a skin-mimicking phantom. The selected microneedle groups (A–D) were constructed based on prior analysis of surface area (SA), volume (Vol), average cross-sectional area (S_Avg), and minimum neck cross-sectional area (S_Min), which were quantified via the Parametric Geometry-Driven Design Platform. Group B served as the control, whereas Group D was the final proposed design, featuring an internal cavity and three barbs.

Group A, whose neck thickness is 62.5% of the microneedle body base, showed the highest axial strength (3.29 ± 0.63 N), indicating that large S_Min affects high resistance against axial compression. Group B, with a neck thickness of 50% of the microneedle body base, exhibited weaker axial performance (1.87 ± 0.32 N), demonstrating that reduced S_Min weakens axial compression resistance. Group C, with a cavity inserted in the middle of the neck when the neck thickness is 50% of the microneedle body base, showed significant structural degradation (1.00 ± 0.09 N). This result shows that reducing both S_Min and S_Avg simultaneously impairs axial compression resistance. However, Group D, which integrates barbs into the cavity-based design, achieved a partial recovery of axial strength (2.13 ± 0.51 N). This result indicates that barbs increase S_Avg without affecting S_Min, thereby increasing split efficiency (S_Avg/S_Min) and providing mechanical reinforcement.

In the shear compression test in Figure 6c, Group A showed the largest detachment force (344.44 ± 47.17 mN), indicating that the thick neck greatly strengthens shear pressure durability. Group B showed a value of (172.90 ± 27.14 mN), indicating that reducing the diameter of the narrow neck was significant in increasing the shear pressure separation efficiency. Furthermore, Group C with the cavity inserted showed less than half (70.23 ± 31.03 mN) of Group B, and in this regard, we observed that the strategy of introducing a cavity in the narrow neck helps the shear pressure separation strategy. Group D with barb structure showed improved axial pressure durability in the axial pressure test; however, it showed a similar value (73.92 ± 34.77) to that of Group C (70.23 ± 31.03 mN) in the shear compression test. Force–displacement curves also revealed similar fracture behaviors between Groups C and D, indicating that repeatedly generated barbs in the axial direction do not interfere with shear separation. Our proposal shows that geometric anisotropy can be enhanced through parameter-based design structure adjustment. This feature increases the microneedle’s design flexibility, enabling axial durability while maintaining lateral separation performance. Consequently, Group D, with high S_Avg and low S_Min, showed improved separation efficiency in mechanical and separation tests, as predicted, which assures the high skin phantom penetration strength with easy separability in comparison with conventional candlelight-shaped microneedles (Group B). However, our study’s concept has the limitation of using non-dissolvable resin and the test was carried out with a parafilm skin phantom. For clinical applications, biodegradable materials would need to replace the current resin, and biological experiments should accompany the material change. But still, the PRISM-generated high-strength separable microneedle design presented effective and durable embedding. We believe that these insights can contribute to the design of next-generation microneedle devices and pave the way for their translation into clinical applications.

## Figures and Tables

**Table 1:** Parametric configurations for microneedle groups.

	Needle_Tip_Angle	Narrowed_Neck_Ratio	Barb_n	Cavity_Scale_x and y	Cavity_Scale_z
Group A	30°	62.5%	No barb	No cavity	No cavity
Group B	30°	50%	No barb	No cavity	No cavity
Group C	30°	50%	No barb	60 µm	80 µm
Group D	30°	50%	3	60 µm	80 µm

**Table 2:** Dimensional accuracy of Group D microneedles compared to CAD specifications.

Structure	Measured Mean	99% CI	CAD Value (µm)	Relative Error (%)
Microneedle Height (µm)	700.07	4.54	700	0.01
Separation Zone Height (µm)	102.3	2.69	100	2.3
Distance to First Barb (µm)	325.62	2.57	325	0.19
1st Barb Diameter (µm)	314.26	10.52	328	4.19
2nd Barb Diameter (µm)	314.03	4.68	328	4.26
3rd Barb Diameter (µm)	319.08	3.17	328	2.72
Max Diameter before Neck (µm)	321.69	5.65	328	1.92
Narrowed Neck Diameter (µm)	161.65	3.26	164	1.43
Cavity XY Diameter (µm)	119.68	1.35	120	0.27

## Data Availability

The original contributions presented in this study are included in the article/Supplementary Material. Further inquiries can be directed to the corresponding authors.

## Supplementary Materials

The following supporting information can be downloaded at: https://www.mdpi.com/article/10.3390/mi16070726/s1.
