# Supplementary material for: Parametric Rule-Based Intelligent System (PRISM) for Design and Analysis of High-Strength Separable Microneedles
Source: Micromachines (Basel). 2025 Jun 21;16(7):726. doi: 10.3390/mi16070726 (PMC12298898; doi:10.3390/mi16070726)
Supplement: Supplementary file 1 [file micromachines-16-00726-s001.zip › Supplementary Note S1.pdf]

# Supplementary Note for Parametric Rule-based Intelligent System (PRISM) for Design and Analysis of High-Strength Separable Microneedles

**Equation S1. Base radius from tip angle**

$$r_{\text{bottom}} = r_{\text{tip}} + h \cdot \tan(\theta_{\text{tip}})$$

$r_{\text{bottom}}$ corresponds to the maximum diameter of the upper needle body and is also the radius of the base of a single bar structure. $r_{\text{bottom}}$ is the floor radius of the upper body of the microneedle, $r_{\text{tip}}$ is the end radius of the microneedle, and $h$ is the height of the microneedle upper body. $\theta_{\text{tip}}$ is the angle of the microneedle upper body. In equation S1, the tip radius of the microneedle upper body and the tip radius of the single bar structure were fixed to 5 $\mu m$, so that the both of microneedle upper body and the barb had a tip of 10 $\mu m$ in diameter.

**Equation S2. Narrowed neck radius**

$$r_{\text{neck}} = \text{narrowed neck ratio} \times r_{\text{bottom}}$$

The radius of the narrowed neck was computed by multiplying the microneedle's upper body's bottom radius ($r_{\text{bottom}}$) by the user-defined narrowed neck ratio, This ratio defines the radius size of narrowed neck ($r_{\text{neck}}$) which plays a critical role in balancing mechanical resistance and separability.

**Equation S3. Barb allocation zone height**

$$h_{\text{barb-zone}} = h_{\text{body}} - d_{\text{offset}}$$

Barbed structures were modeled as identical truncated cones arranged vertically along the lower shaft. The total height allocated for barb placement ($h_{\text{barb-zone}}$) is defined as the needle body height minus an offset distance ($d_{\text{offset}}$) from the needle tip to the first barb position, The offset was fixed at 0.05 mm to ensure reproducibility and to prevent distortion of needle tip during printing. This offset value was determined based on the 5 μm slicing resolution of the DLP printer and implemented in the Python script.

**Equation S4. Height of each barb**

$$h_{\text{barb}} = h_{\text{barb-zone}} \cdot 0.5$$

The height of each barb was set as half of the total barb allocation zone ($h_{\text{barb-zone}}$), This ensured that the total barb zone height corresponds exactly to twice the height of a single barb, serving as the basis for determining individual barb dimensions.

**Equation S5. Barb spacing**

$$\text{barb spacing} = \left(\frac{2-n}{n-1}\right) \cdot h_{\text{barb}}$$

To evenly distribute barb spacing within the designated zone, the spacing between adjacent barbs was calculated by using the equation s5. $h_{\text{barb}}$ is the height of the single barb structure and $n$ is the number of barb.

**Equation S6. Barb half-apex angle**

$$\theta_{\text{barb}} = \tan^{-1}\left(\frac{r_{\text{bottom}} - r_{\text{top}}}{h_{\text{barb}}}\right)$$

The half-apex angle of each barb was defined as the arctangent of the radial difference over the barb height, This formula yields the half-apex angle; the full apex angle of the barb is twice this value. $\theta_{\text{barb}}$ is the angle of the barb structures and both of $r_{\text{bottom}}$ and of $r_{\text{top}}$ in equation S6 are the parameters of barb structure.

Equations (S1) through (S6) were applied consistently to ensure precise and uniform microneedle geometry: (S1) defines the body taper, (S2) calculates the narrowed neck radius, (S3) determines the effective barb allocation zone, (S4) sets the individual barb height, (S5) ensures uniform barb spacing, and (S6) defines the barb cone angle for structural validation.
